# Comparative efficacy of postoperative adjuvant transcatheter arterial chemoembolization and hepatic artery infusion chemotherapy in patients with BCLC stage 0-B hepatocellular carcinoma at high risk of recurrence following radical resection

**DOI:** 10.3389/fphar.2025.1657794

**Published:** 2025-09-19

**Authors:** Xu Feng, Yupei Ao, Jiarui Liu, Jue Yuan, Zhengrong Shi, Chengjia Tang

**Affiliations:** ^1^ Department of Hepatobiliary Surgery, The Affiliated Yongchuan Hospital of Chongqing Medical University, Chongqing, China; ^2^ Department of Hepatobiliary Surgery, The First Affiliated Hospital of Chongqing Medical University, Chongqing, China; ^3^ Health Screening Centre, Chongqing Western Hospital, Chongqing, China

**Keywords:** hepatocellular carcinoma, radical resection, postoperative adjuvant, transcatheter arterial chemoembolization, hepatic artery infusion chemotherapy

## Abstract

**Aim:**

This study aims to compare the efficacy of postoperative adjuvant transcatheter arterial chemoembolization (PA-TACE) and postoperative adjuvant hepatic artery infusion chemotherapy (PA-HAIC) in patients with BCLC Stage 0-B Hepatocellular Carcinoma (HCC) at high risk of recurrence following radical resection.

**Methods:**

This study retrospectively evaluated HCC patients who underwent radical liver resection (LR) at two clinical centers between 1 January 2018, and 31 December 2024. The recurrence-free survival (RFS) and overall survival (OS) were compared among three groups: those who received LR alone, PA-TACE, and PA-HAIC. Propensity score matching (PSM) was applied to minimize inter-group differences and further validate the findings.

**Results:**

A total of 609 patients with high-risk recurrence following radical resection of HCC were included in this study. After PSM, both PA-TACE and PA-HAIC significantly improved median RFS (mRFS) and median OS (mOS) compared with LR alone (mRFS for the LR, PA-TACE, and PA-HAIC groups was 16.5 months, 39.0 months, and 46.0 months, respectively; mOS was 54.0 months, 68.0 months, and not reached for PA-HAIC, respectively). Furthermore, patients treated with PA-HAIC achieved superior mRFS as well as higher 1-year, 2-year, and 4-year RFS rates compared with those treated with PA-TACE. Similarly, PA-HAIC was associated with a significantly longer mOS and a higher 4-year OS rate than PA-TACE. In the construction of the RFS nomogram, the C-indexes for the training and validation cohorts were 0.802 and 0.799, respectively, demonstrating good predictive ability.

**Conclusion:**

In HCC patients with high-risk recurrence following radical resection, PA-HAIC significantly improves RFS compared to PA-TACE, but only in patients with MVI, tumor diameter ≥5 cm, or multiple tumors.

## 1 Introduction

Primary liver cancer is the sixth most common cancer globally and the second leading cause of cancer-related deaths, with a particularly high prevalence in resource-limited developing countries. Hepatocellular carcinoma (HCC) accounts for approximately 75%–85% of all primary liver cancer cases (Kocarnik et al., 2022; Bray et al., 2018). In China, HCC is the fifth most common cancer and the second leading cause of cancer-related deaths (Zheng et al., 2024). For HCC treatment, radical methods such as ablation, radical liver resection (LR), and liver transplantation are the primary choices (Benson et al., 2021; Sun et al., 2022; European Association for the Study of the Liver, 2018). However, due to the inherent heterogeneity of HCC, there still remains a high recurrence rate even after curative resection. According to statistics, the recurrence rate within 5 years after radical resection remains as high as 50%–70%. Compared to patients without recurrence, the 5-year survival rate of patients with HCC recurrence is reduced by approximately 24% (Bruix et al., 2014; Kim et al., 2020; Tsilimigras et al., 2020).

It is widely believed that multiple tumors or satellite lesions, tumor diameter ≥5 cm, vascular invasion, and poor differentiation are high-risk recurrent factors recurrence (HRRFs) after radical resection of HCC (Guo et al., 2023; Liu et al., 2020; Wakayama et al., 2017; Erstad and Tanabe, 2019). Previous studies demonstrated that the 4-month recurrence rate of patients with portal vein tumor thrombus (PVTT) was 78.3%, the 1-year recurrence rate of patients with microvascular invasion (MVI) was nearly 50%, while the 6-month recurrence rate of patients with multiple tumors was 60% (Choi et al., 2011; Chen et al., 2021; Kim et al., 2006). The 5-year survival rates for HCC patients with left/right PVTT, MVI, and multiple tumors were only 32.9%, 33.3%, and 31.9%, respectively (Chen et al., 2021; Orimo et al., 2022; Kokudo et al., 2016). Therefore, reducing the early recurrence rate after radical resection is crucial to improve the long-term survival of HCC patients with HRRFs.

Numerous studies have demonstrated that postoperative adjuvant transcatheter arterial chemoembolization (PA-TACE) and hepatic artery infusion chemotherapy (PA-HAIC) after LR have good efficacy in HCC patients with HRRFs (Yang et al., 2021; Xiang et al., 2024; Hu et al., 2023). Several network meta-analyses have proven that compared to PA-TACE, PA-HAIC after LR is more effective in improving overall survival time (OS) and recurrence-free survival time (RFS) (Liu et al., 2021; Feng et al., 2023; Pei et al., 2024). Another multicenter retrospective study showed that compared to PA-TACE, PA-HAIC demonstrated greater benefits in preventing tumor recurrence and improving survival in HCC patients with MVI (Wen et al., 2024). Therefore, in this study, we will once again explore the comparison of the efficacy of PA-TACE and PA-HAIC after radical resection in HCC with HRRFs.

## 2 Materials and methods

### 2.1 Patients

This study retrospectively evaluated patients with HCC who underwent LR at the Department of Hepatobiliary Surgery of the First Affiliated Hospital of Chongqing Medical University and the Department of Hepatobiliary Surgery of the Affiliated Yongchuan Hospital of Chongqing Medical University from 1 January 2018 to 31 December 2024. The study was conducted in accordance with the Helsinki Declaration and was approved by the Institutional Ethics Committee of the First Affiliated Hospital of Chongqing Medical University (K2014-039-01) and the Affiliated Yongchuan Hospital of Chongqing Medical University (2025EC0058). As this study is retrospective, obtaining further informed consent from patients was not required.

Patient inclusion criteria were as follows: (1) histologically confirmed HCC with negative margins (R0 resection); (2) presence of recurrence risk factors, including multiple tumors or satellite lesions, tumor diameter ≥5 cm, poor histological differentiation, or MVI; (3) tumor staging within Barcelona Clinic Liver Cancer (BCLC) stage 0–B; (4) no anti-tumor therapy administered prior to resection; and (5) no history of other malignancies. Exclusion criteria: (1) incomplete clinical data or follow-up; (2) non-radical resection (R1 resection) or preoperative imaging indicating portal vein invasion or extrahepatic metastasis; (3) postoperative pathological confirmation of non-HCC such as cholangiocarcinoma; (4) coexistence of other malignant tumors or severe organ diseases, including brain, heart, and lung diseases; (5) absence of high-risk factors for recurrence; (6) received postoperative treatments other than PA-TACE or PA-HAIC; (7) treatment crossover between PA-TACE and PA-HAIC; (8) Recurrence or death within 30 days post-surgery. Two senior pathologists interpreted and confirmed the pathological diagnosis. MVI was diagnosed when cancer cell clusters were observed in the vascular lumen lined with endothelial cells, typically seen in the small branches of the portal vein in the peritumoral liver tissue or the blood vessels within the tumor capsule (Evidence-based practice guidelines for standardized, 2015).

### 2.2 Radical liver resection and postoperative adjuvant therapy

All patients underwent routine preoperative examinations to assess tumor diameter, BCLC stage, resectability, residual liver volume, and extent of cirrhosis. The hepatectomy method contains non-anatomical resection and anatomical resection. Anatomical hepatectomy is the complete resection of the segment of liver with the tumor or the segment of liver limited by the branches of the portal vein of the tumor. Non-anatomical hepatectomy is the resection of the tumor and part of the non-tumor liver parenchyma (Zhou et al., 2001; Makuuchi et al., 1985). Radical LR was defined as the complete removal of all detected tumors without involving any major branch of the portal or hepatic veins, without invasion of adjacent organs and without lymph node or distant metastasis, and tumor-free margins confirmed by histopathology (European Association for the Study of the Liver, 2018).

The selection of PA-TACE or PA-HAIC treatment regimens is typically made by attending physicians at the associate chief physician level or higher, based on the patient’s clinical data, the physician’s clinical experience, and relevant research. Recommendations and associated risks are then communicated to the patient or their family, with the final treatment decision made by the patient or their family. Meanwhile, all patients with HBV infection need to be treated with antiviral drugs.

PA-TACE: Patients are typically scheduled for PA-TACE approximately 4–6 weeks following radical resection. Prior to the procedure, a series of tests are conducted, including measurement of alpha-fetoprotein (AFP), and enhanced upper abdominal computed tomography (CT) or magnetic resonance imaging (MRI). A hepatic artery catheter was inserted via the femoral artery using the Seldinger technique, and the presence of intrahepatic tumor staining was evaluated by digital subtraction angiography (DSA) or CT angiography. In the absence of tumor staining, chemotherapy drugs (one or more of the following: fluorouracil, anthracyclines, or platinum-based agents) and embolic agents (like lipiodol and/or gelatin sponge) are administered via the catheter to the liver segment containing the tumor, based on comprehensive evaluation of the patient’s body surface area, physical condition, and residual liver volume. After administration, the catheter is removed, and the puncture site is compressed and bandaged, after which the patient is returned to the ward for recovery. Patients are allowed to mobilize the right lower limb after 8 h. If subsequent routine blood tests and liver function tests show no significant abnormalities, the patient is discharged. The interval between two PA-TACE cycles is typically 4–5 weeks (Raoul et al., 2019).

PA-HAIC: PA-HAIC follows a schedule and procedure similar to that of PA-TACE. After positioning the hepatic artery catheter at the predetermined site, the patient is transferred to the ward. The catheter is connected to an infusion pump in the ward, which continuously administers the following chemotherapy drugs: oxaliplatin at 85 mg/m^2^ over 0–3 h on day 1; leucovorin at 400 mg/m^2^ over 3–4.5 h on day 1; fluorouracil at 400 mg/m^2^ over 4.5–6.5 h on day 1; and fluorouracil at 2,400 mg/m^2^ over 46 h from day 1 to day 3. During the infusion, the patient’s movement of the right lower limb is almost completely restricted. After the chemotherapy is completed, the catheter is removed, and the puncture site is compressed and bandaged. The patient can move their right lower limb after 8 h. The interval between two PA-HAIC cycles is also set at 4–5 weeks.

### 2.3 Follow up and outcomes

All patients were followed up every 1–2 months for 6 months after discharge from the hospital and every 3–6 months thereafter. During the follow-up period, each patient received routine blood tests, liver function tests, AFP, and abdominal ultrasound. If recurrence was suspected, enhanced CT or enhanced MRI was used to confirm the diagnosis. Recurrence was defined as any tumor nodule confirmed by two imaging studies or puncture biopsy. The primary endpoint of the study is RFS, defined as the time from LR to the diagnosis of tumor recurrence, or the end of the follow-up period. The secondary endpoint is OS, defined as the time from LR to death, or the end of the follow-up period. All patients are followed until 1 April 2025, loss to follow-up, or death.

### 2.4 Propensity score matching

Propensity score matching (PSM) analysis was conducted to minimize the uneven distribution of covariates between the three groups. In the PSM analysis, matching was performed using the nearest neighbor method with a variable ratio and a caliper width of 0.02, and the matching criteria included age, gender, BCLC stage, presence of hepatitis, presence of cirrhosis, AFP level, ALBI grade, PALBI grade, hemoglobin, NLR, PLR, tumor number, tumor diameter, MVI expression, and degree of differentiation, with covariate balance assessed using standardized mean differences (SMD), where SMD <0.1 indicated adequate balance. Following the PSM, we conducted an inverse-probability-of-treatment-weighting (IPTW) sensitivity analysis to assess result robustness.

### 2.5 Statistical analysis

The Shapiro-Wilk test was used to test the normality of continuous variables, and the independent samples t-test was used to detect continuous data that followed the normal distribution, expressed as the mean ± standard deviation. The Mann-Whitney U test was used to detect continuous data that were not normally distributed, expressed as median (interquartile range, IQR). Categorical data were detected using the chi-squared test, and expressed as numbers (n) and proportions (%). Univariate and multivariate analyses were performed in Cox risk models to identify independent prognostic factors. Survival analyses were performed using the Kaplan–Meier (K-M) method. Risk factors identified from the multivariate COX regression analysis were incorporated into a nomogram to develop a predictive model for high-risk recurrence in HCC. Model fit was assessed with a calibration plot by means of 300 boot-strap resamples. The predictive accuracy of the nomogram was evaluated by analyzing receiver operating characteristic (ROC) curves at 1, 2, 3, and 4 years. The area under the curve (AUC) from 1 to 48 months was measured using time-dependent AUC and the time-dependent concordance index (C-index) to assess predictive accuracy at various time points. All statistical analyses were two-tailed, with a P-value <0.05 considered statistically significant. Analyses were conducted using SPSS 27.0, R software (version 4.5.0, http://www.r-project.org), and DecisionLinnc software (version V1.0, https://www.statsape.com/).

## 3 Result

### 3.1 Baseline patient characteristics

**FIGURE 1:**
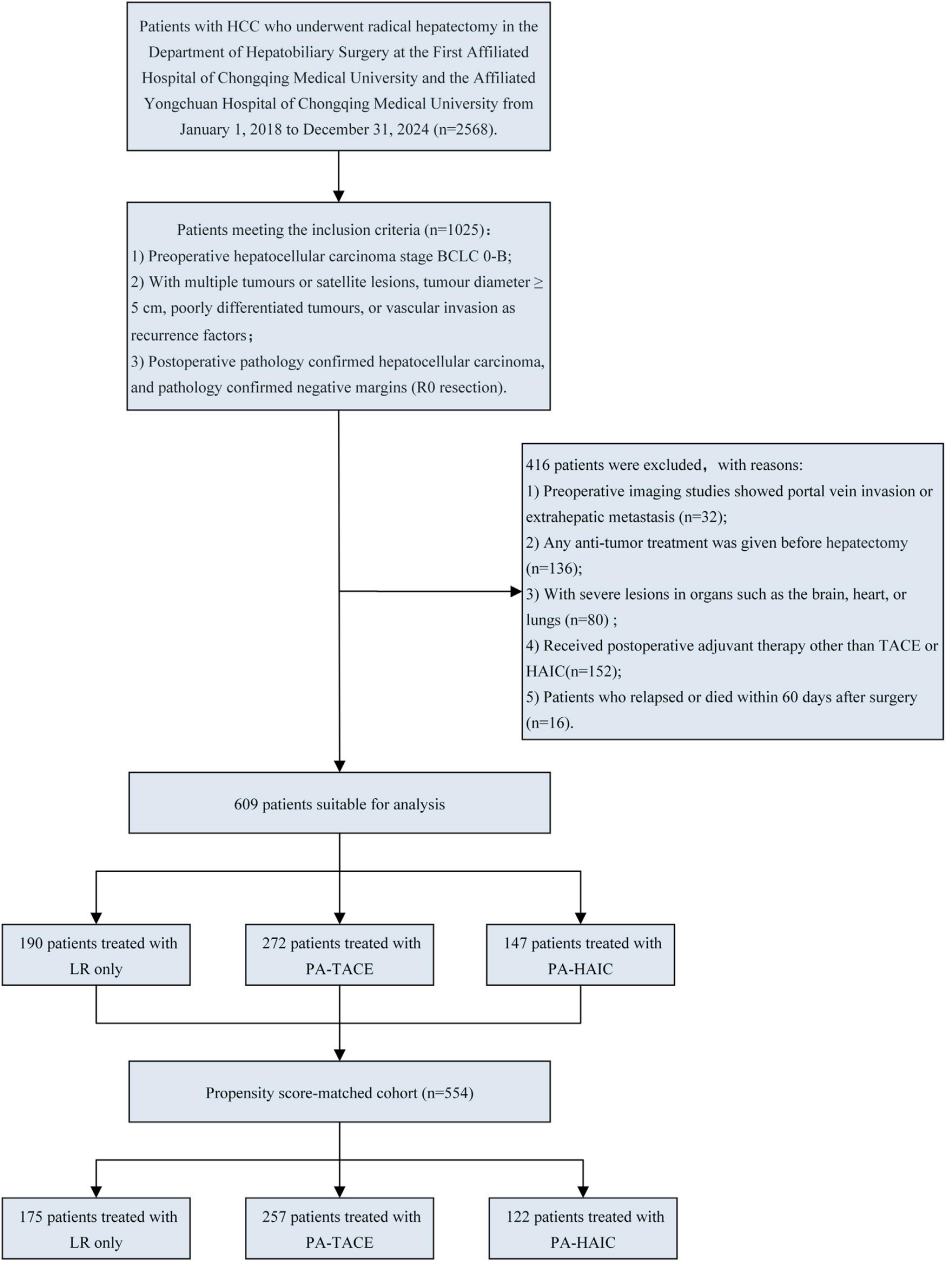
Flowchart of patient selection. LR, liver resection; PA, Postoperative adjuvant; TACE, transcatheter arterial chemoembolization; HAIC, Hepatic artery perfusion chemotherapy; BCLC, Barcelona Clinic Liver Cancer.

After screening, a total of 609 eligible patients were included in this study. The patient selection process is shown in Figure 1.

In the entire cohort, patients had an age range of 56.47 ± 10.56 years, with 529 male patients (86.86%) and 80 female patients (13.14%). Among them, 190 patients (31.20%) did not receive any adjuvant therapy, 272 patients (44.66%) underwent PA-TACE, and 147 patients (24.14%) received PA-HAIC. In the PSM cohort, the average age was 56.75 ± 10.43 years, with 483 male patients (87.18%) and 71 female patients (12.82%). Within this group, 175 patients (31.59%) did not receive any adjuvant therapy, 257 patients (46.39%) underwent PA-TACE, and 122 patients (22.02%) received PA-HAIC. The baseline characteristics for both the entire cohort and the matched cohort are summarized in Tables 1 and 2.

**TABLE 1:** Baseline characteristics of HCC patients with HRRFs after LR.

Characteristics		The entire cohort					The PSM cohort				
		LR (*n =* 190)	PA-TACE (*n =* 272)	PA-HAIC (*n =* 147)	SMD	p	LR (*n =* 175)	PA-TACE (*n =* 257)	PA-HAIC (*n =* 122)	SMD	p
Age, years		56.98 ± 11.63	55.93 ± 9.77	56.80 ± 10.53	0.067	0.523	57.03 ± 11.50	56.11 ± 9.71	57.70 ± 10.28	0.102	0.350
Age, n (%)	≤56 years	91 (47.89)	141 (51.84)	72 (48.98)	0.053	0.687	86 (49.14)	133 (51.75)	55 (45.08)	0.089	0.477
	>56 years	99 (52.11)	131 (48.16)	75 (51.02)			89 (50.86)	124 (48.25)	67 (54.92)		
Gender (%)	Male	167 (87.89)	233 (85.66)	129 (87.76)	0.044	0.752	153 (87.43)	225 (87.55)	105 (86.07)	0.029	0.916
	Female	23 (12.11)	39 (14.34)	18 (12.24)			22 (12.57)	32 (12.45)	17 (13.93)		
Hepatitis, n (%)	HBV	155 (81.58)	216 (79.41)	124 (84.36)	0.114	0.842	140 (80.00)	207 (80.55)	102 (83.61)	0.109	0.928
	HCV	11 (5.79)	20 (7.35)	8 (5.44)			11 (6.29)	18 (7.00)	6 (4.92)		
	AH	9 (4.74)	17 (6.25)	5 (3.40)			9 (5.14)	14 (5.45)	4 (3.28)		
	No hepatitis	15 (7.89)	19 (6.99)	10 (6.80)			15 (8.57)	18 (7.00)	10 (8.19)		
Liver Cirrhosis yes, n (%)		115 (60.53)	151 (55.51)	89 (60.54)	0.068	0.502	106 (60.57)	148 (57.59)	69 (56.56)	0.054	0.749
AFP, n (%)	<200 ng/mL	122 (64.21)	189 (69.49)	116 (78.91)	0.220	0.013	118 (67.43)	181 (70.43)	94 (77.05)	0.144	0.193
	≥200 ng/mL	68 (35.79)	83 (30.51)	31 (21.09)			57	76 (29.57)	28 (22.95)		
Tumor diameter, cm		4.20 (2.50, 6.00)	4.05 (2.80, 6.10)	4.10 (2.70, 6.50)	0.051	0.766	4.50 (2.70, 6.00)	4.00 (2.75, 6.00)	3.85 (2.48, 6.20)	0.049	0.721
Tumor diameter, n (%)	<5 cm	113 (59.47)	164 (60.29)	80 (54.42)	0.079	0.487	102 (58.29)	162 (63.04)	74 (60.66)	0.065	0.608
	≥5 cm	77 (40.53)	108 (39.71)	67 (45.58)			73 (41.71)	95 (36.96)	48 (39.34)		
Tumor number, n (%)	Solitary	127 (66.84)	204 (75.00)	99 (67.35)	0.120	0.103	124 (70.86)	192 (74.71)	89 (72.95)	0.058	0.675
	Multiple	63 (33.16)	68 (25.00)	48 (32.65)			51 (29.14)	65 (25.29)	33 (27.05)		
BCLC grade, n (%)	0+A	154 (81.05)	220 (80.88)	106 (72.11)	0.142	0.077	142 (81.14)	208 (80.93)	96 (78.69)	0.041	0.847
	B	36 (18.95)	52 (19.12)	41 (27.89)			33 (18.86)	49 (19.07)	26 (21.31)		
Number of interventions	1	-	55 (20.22)	18 (12.24)	-	0.284	-	55 (21.40)	14 (11.48)	-	0.108
	2	-	89 (32.72)	54 (36.74)			-	80 (31.13)	44 (36.07)		
	3	-	78 (28.68)	41 (27.89)			-	76 (29.57)	33 (27.05)		
	4	-	30 (11.03)	21 (14.29)			-	27 (10.51)	18 (14.75)		
	5	-	20 (7.35)	13 (8.84)			-	19 (7.39)	13 (10.65)		

PSM, Propensity score matching; LR, liver resection; PA, postoperative adjuvant; TACE, transcatheter arterial chemoembolization; HAIC, hepatic artery perfusion chemotherapy; HBV, Hepatitis B virus; HCV, Hepatitis C virus; AH, alcoholic hepatitis; AFP, Alpha-fetoprotein; BCLC, barcelona clinic liver cancer. In the PSM cohort, the SMDs were generally ≤0.1, indicating minimal differences in baseline characteristics between the groups.

**TABLE 2:** Surgery-related variables of HCC patients with HRRFs after LR.

Characteristics		The entire cohort					The PSM cohort				
		LR (*n =* 190)	PA-TACE (*n =* 272)	PA-HAIC (*n =* 147)	SMD	p	LR (*n =* 175)	PA-TACE (*n =* 257)	PA-HAIC (*n =* 122)	SMD	p
Hemoglobin, n (%)	≤140 g/L	98 (51.58)	135 (49.63)	65 (44.22)	0.098	0.389	88 (50.29)	123 (47.86)	56 (45.90)	0.059	0.750
	>140 g/L	92 (48.42)	137 (50.37)	82 (55.78)			87 (49.71)	134 (52.14)	66 (54.10)		
Child-pugh score	5	152 (80.00)	241 (88.60)	124 (84.35)	0.651	0.038	145 (82.86)	228 (88.72)	107 (88.70)	0.112	0.198
	6	38 (20.00)	31 (11.40)	23 (15.65)			30 (17.14)	29 (11.28)	15 (12.30)		
NLR, n (%)	<2.4	86 (45.26)	135 (49.63)	70 (47.62)	0.058	0.656	77 (44.00)	132 (51.36)	56 (45.90)	0.098	0.287
	≥2.4	104 (54.74)	137 (50.37)	77 (52.38)			98 (56.00)	125 (48.64)	66 (54.10)		
PLR, n (%)	<145	136 (71.58)	180 (66.18)	111 (75.51)	0.138	0.122	123 (70.29)	177 (68.87)	88 (72.13)	0.048	0.808
	≥145	54 (28.42)	92 (33.82)	36 (24.49)			52 (29.71)	80 (31.13)	34 (27.87)		
ALBI grade, n (%)	1	130 (68.42)	188 (69.12)	94 (63.95)	0.073	0.540	124 (70.86)	179 (69.65)	81 (66.39)	0.064	0.705
	2	60 (31.58)	84 (30.88)	53 (36.05)			51 (29.14)	78 (30.35)	41 (33.61)		
PALBI grade, n (%)	1	126 (66.31)	181 (66.54)	90 (61.22)	0.156	0.311	120 (68.57)	178 (69.26)	79 (64.75)	0.065	0.936
	2	55 (28.95)	85 (31.25)	54 (36.74)			51 (29.14)	73 (28.40)	40 (32.79)		
	3	9 (4.74)	6 (2.21)	3 (2.04)			4 (2.29)	6 (2.34)	3 (2.46)		
Total protein, n (%)	<70	83 (43.68)	135 (49.63)	70 (47.62)	0.080	0.450	74 (42.29)	129 (50.19)	59 (48.36)	0.106	0.261
	≥70	107 (56.32)	137 (50.37)	77 (52.38)			101 (57.71)	128 (49.81)	63 (51.64)		
ALT, n (%)	<34 U/L	92 (48.42)	137 (50.37)	68 (46.26)	0.055	0.723	85 (48.57)	130 (50.58)	59 (48.36)	0.030	0.885
	≥34 U/L	98 (51.58)	135 (49.63)	79 (53.74)			90 (51.43)	127 (49.42)	63 (51.64)		
AST, n (%)	<34 U/L	93 (48.95)	138 (50.74)	66 (44.90)	0.078	0.520	87 (49.71)	130 (50.58)	57 (64.72)	0.052	0.779
	≥34 U/L	97 (51.05)	134 (49.26)	81 (55.10)			88 (50.29)	127 (49.42)	65 (53.28)		
ALP, n (%)	<90 U/L	106 (55.79)	146 (53.68)	77 (52.38)	0.046	0.833	98 (56.00)	139 (54.09)	69 (56.56)	0.033	0.876
	≥90 U/L	84 (44.21)	126 (46.32)	70 (47.62)			77 (44.00)	118 (45.91)	53 (43.44)		
Differentiation, n (%)	Low	49 (25.79)	91 (33.46)	44 (29.93)	0.112	0.215	47 (26.86)	87 (33.85)	38 (31.15)	0.102	0.304
	High and/or moderate	141 (74.21)	181 (66.54)	103 (70.07)			128 (73.14)	170 (66.15)	84 (68.85)		
MVI	Positive	115 (60.53)	176 (64.71)	107 (72.79)	0.164	0.084	113 (64.57)	166 (64.59)	82 (67.21)	0.037	0.865
	Negative	75 (39.47)	96 (35.29)	40 (27.21)			62 (35.43)	91 (35.41)	40 (32.79)		
Resection pattern, n (%)	Anatomic	146 (76.84)	221 (81.25)	112 (76.19)	0.083	0.366	135 (77.14)	209 (81.32)	95 (77.87)	0.069	0.526
	Nonanatomic	44 (23.16)	51 (18.75)	35 (23.81)			40 (22.86)	48 (18.68)	27 (22.13)		
Blood transfusion yes, n (%)		23 (12.11)	27 (9.93)	17 (11.56)	0.046	0.760	21 (12.00)	27 (10.51)	12 (9.84)	0.046	0.818
Hemorrhage, mL		300 (150, 500)	200 (100, 400)	300 (150, 500)	0.099	0.121	300 (150, 500)	200 (100, 400)	292.50 (104, 400)	0.058	0.291
Operating time, minutes		240 (190, 310)	260 (200, 320)	260 (190, 320)	0.118	0.330	240 (190, 310)	260 (200, 320)	250 (187, 320)	0.113	0.365
Resection margin ≥ 1 cm, n (%)		190 (100.00)	272 (100.00)	147 (100.00)	-	1.000	175 (100.00)	257 (100.00)	122 (100.00)	-	1.000

PSM, Propensity score matching; LR, liver resection; PA, postoperative adjuvant; TACE, transcatheter arterial chemoembolization; HAIC, hepatic artery perfusion chemotherapy; NLR, Neutrophil-Lymphocyte Ratio; PLR, Platelet-Lymphocyte Ratio; ALBI, albumin-bilirubin, ALBl = (log10 total bilirubin*0.66) + (albumin * −0.085), ALBI, grade 1, ≤ −2.60; ALBI, grade 2, −2.60 ∼ −1.39; PALBI = (2.02*log10 total bilirubin)-0.37*(log10 total bilirubin)^2^-(0.04*albumin) - (3.48*log10 platelet) + 1.01*(log10 platelet)^2^. ALT, alanine aminotransferase; AST, alanine aminotransferase; ALP, alkaline phosphatase; PALBI, grade 1, ≤ −2.53; ALBI, grade 2, −2.53 ∼ −2.09; PALBI, grade 3, >2.09; MVI, microvascular invasion. In the PSM, cohort, the SMDs, were generally ≤0.1, indicating minimal differences in baseline characteristics between the groups.

### 3.2 Recurrence-free survival time

In the entire cohort, 157 patients (82.63%) in the LR group experienced recurrence, while 191 patients (70.22%) in the PA-TACE group and 96 patients (65.31%) in the PA-HAIC group experienced recurrence. The median RFS (mRFS) for the LR, PA-TACE, and PA-HAIC groups were 16.00 months (95% CI, 14.20–17.80 months), 38.50 months (95% CI, 36.81–40.19 months), and 42.00 months (95% CI, 38.21–45.79 months), respectively, with statistically significant differences among the three groups. The 1-year, 2-year, and 3-year RFS rates for patients in the LR group were significantly lower than those in the PA-TACE and PA-HAIC groups. Additionally, the 1-year, 2-year, and 4-year RFS rates for the PA-TACE group were significantly lower than those for the PA-HAIC group, although there was no significant difference in the 3-year RFS rate between the PA-TACE and PA-HAIC groups.

**FIGURE 2:**
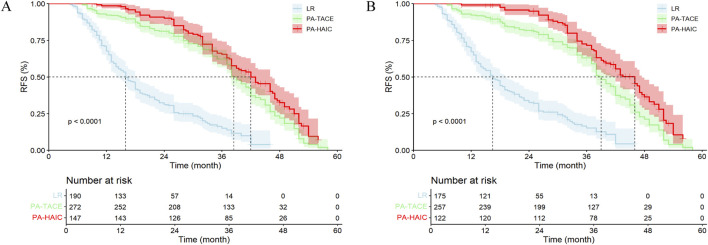
The Kaplan-Meier survival analysis of HCC patients RFS between different postoperative adjuvant therapy. **(A)** The entire cohort; **(B)** The PSM cohort; RFS, recurrence-free survival time; LR, liver resection; PA, Postoperative adjuvant; TACE, transcatheter arterial chemoembolization; HAIC, Hepatic artery perfusion chemotherapy. **(A)** mRFS for the LR, PA-TACE, and PA-HAIC groups were 16.00 m, 38.50 m, and 42.00 m, respectively; **(B)** mRFS for the LR, PA-TACE, and PA-HAIC groups were 16.00 m, 38.50 m, and 42.00 m, respectively.

In the PSM cohort, 143 patients (81.71%) in the LR group, 181 patients (70.43%) in the PA-TACE group, and 78 patients (63.93%) in the PA-HAIC group experienced recurrence. The differences in 1-year, 2-year, 3-year, and 4-year RFS rates and mRFS among the three groups were consistent with those observed in the entire cohort. Further details are available in Figure 2 and Supplementary Table S1.

### 3.3 Overall survival time

In the entire cohort, 48 patients (25.26%) in the LR group died, compared to 42 patients (15.44%) in the PA-TACE group and 20 patients (13.61%) in the PA-HAIC group. The median OS (mOS) was 52.0 months (95% CI: 49.26–54.75 months) for LR, 68.0 months (95% CI: 62.02–73.98 months) for PA-TACE, and was not reached in the PA-HAIC group. Both PA-TACE and PA-HAIC group significantly longer OS compared to LR group, although no significant difference was observed between PA-TACE and PA-HAIC group. The 3-, 4-, and 5-year OS rates were all significantly lower in the LR group compared with both PA-TACE and PA-HAIC groups, whereas no significant difference was found between the PA-TACE and PA-HAIC groups.

**FIGURE 3:**
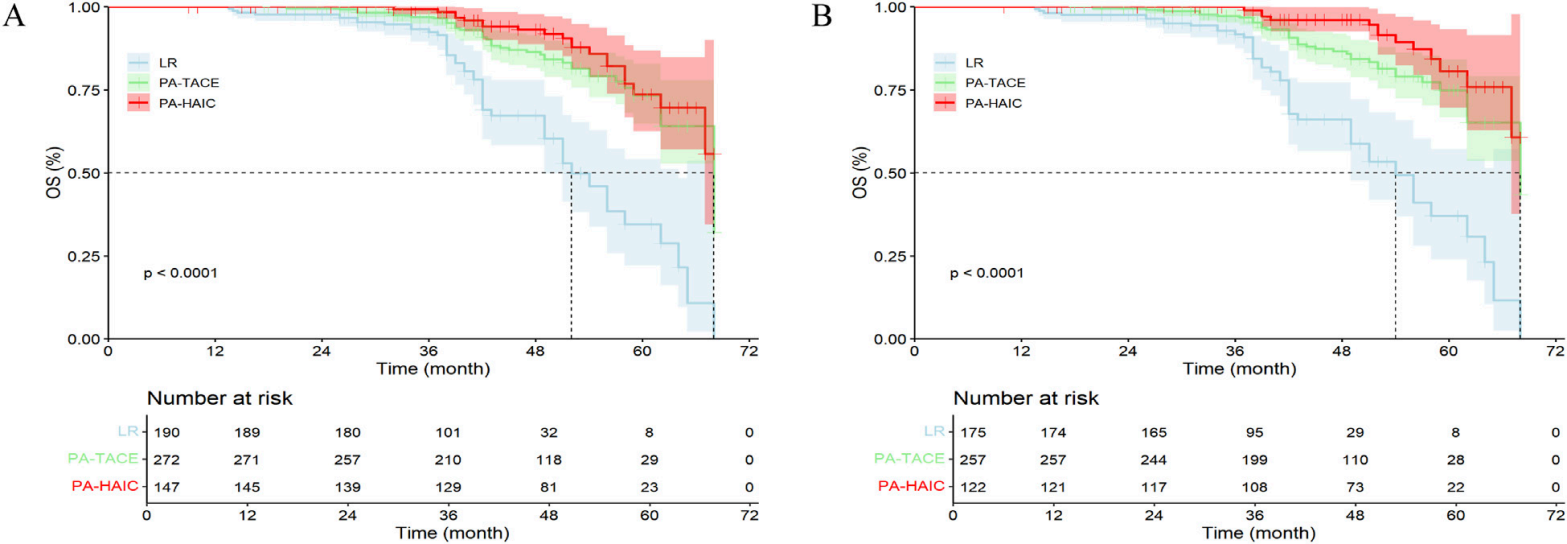
The Kaplan-Meier survival analysis of HCC patients OS between different postoperative adjuvant therapy. **(A)** The entire cohort; **(B)** The PSM cohort; OS, Overall survival time; LR, liver resection; PA, Postoperative adjuvant; TACE, transcatheter arterial chemoembolization; HAIC, Hepatic artery perfusion chemotherapy. **(A)** The mOS for the LR, PA-TACE, and PA-HAIC groups were 52.0 m, 68.0 m, and not reached, respectively; **(B)** The mOS for the LR, PA-TACE, and PA-HAIC groups were 54.0 m, 68.0 m, and not reached, respectively.

In the PSM cohort, the mOS differed significantly among all three groups (54.0 months (95% CI: 48.33–59.67 months) for LR, 68.0 months (95% CI: 58.13–77.89 months) for PA-TACE, and not reached for PA-HAIC). Similarly, the 3-, 4-, and 5-year OS rates for PA-TACE and PA-HAIC groups were significantly higher than those for LR groups. Additionally, PA-HAIC demonstrated a statistically higher 4-year OS rate than PA-TACE, whereas no significant differences were observed between PA-TACE and PA-HAIC at 2-, 3-, or 5- year OS rates. Further details are available in Figure 3 and Supplementary Table S2.

### 3.4 COX regression analysis

Univariate and multivariate Cox regression analyses were performed in both the entire cohort and the PSM cohort. In the entire cohort, the following variables were identified as independent risk factors for RFS: age ≤56 years (1.507, 95% CI 1.244–1.824), tumor diameter ≥5 cm (2.348, 95% CI 1.909–2.887), multiple tumors (2.806, CI 1.997–3.982), poor differentiation (2.478, 95% CI 2.002–3.067), and MVI positivity (2.517, 95% CI 2.005–3.159). In contrast, compared with LR, both PA-TACE (0.175, 95% CI 0.137–0.223) and PA-HAIC (0.120, 95% CI 0.090–0.160) were identified as independent protective factors for RFS. Similarly, for OS, independent risk factors included age ≤56 years (1.794, 95% CI 1.169–2.754), tumor diameter ≥5 cm (2.203, 95% CI 1.415–3.431), multiple tumors (4.372, 95% CI 2.146–8.907), poor differentiation (2.944, 95% CI 1.909–4.541), and MVI positivity (2.095, 95% CI 1.198–3.661). Again, PA-TACE (0.261, 95% CI 0.167–0.410) and PA-HAIC (0.163, 95% CI 0.093–0.286) were shown to be independent protective factors for OS. Consistent findings were observed in the PSM cohort, reinforcing the reliability of the results. Detailed statistical outcomes are summarized in Tables 3 and 4 and Supplementary Table S3.

**TABLE 3:** Multivariate analysis of RFS in the entire cohort and the PSM cohort.

Characteristics		The entire cohort		The PSM cohort	
		HR (95% CI)	p	HR (95% CI)	p
Type of treatment	LR	Reference	-	Reference	-
	PA-TACE	0.175 (0.137, 0.223)	<0.001	0.181 (0.141, 0.234)	<0.001
	PA-HAIC	0.120 (0.090, 0.160)	<0.001	0.113 (0.083, 0.155)	<0.001
Age, yr (≤56 vs. >56)		1.507 (1.244, 1.824)	<0.001	1.436 (1.175, 1.757)	<0.001
AFP, ng/mL (≥200 vs. < 200)		1.036 (0.833, 1.289)	0.750	1.038 (0.820, 1.315)	0.755
Tumor diameter, cm (≥5 vs. <5)		2.348 (1.909, 2.887)	<0.001	2.358 (1.893, 2.936)	<0.001
Tumor number (Multiple vs. Single)		2.806 (1.997, 3.982)	<0.001	2.468 (1.674, 3.640)	<0.001
BCLC (B vs. 0+A)		0.748 (0.508, 1.102)	0.142	0.820 (0.532, 1.262)	0.366
Differentiation (Low vs. High and/or moderate)		2.478 (2.002, 3.067)	<0.001	2.471 (1.974, 3.093)	<0.001
MVI (Positive vs. Negative)		2.517 (2.005, 3.159)	<0.001	2.643 (2.073, 3.368)	<0.001

PSM, Propensity score matching; LR, liver resection; PA, postoperative adjuvant; TACE, transcatheter arterial chemoembolization; HAIC, hepatic artery perfusion chemotherapy; BCLC, barcelona clinic liver cancer; MVI, microvascular invasion.

**TABLE 4:** Multivariate analysis of OS in the entire cohort and the PSM cohort.

Characteristics		The entire cohort		The PSM cohort	
		HR (95% CI)	p	HR (95% CI)	p
Type of treatment	LR	Reference	-	Reference	-
	PA-TACE	0.261 (0.167, 0.410)	<0.001	0.284 (0.179, 0.449)	<0.001
	PA-HAIC	0.163 (0.093, 0.286)	<0.001	0.137 (0.071, 0.264)	<0.001
Age, yr (≤56 vs. >56)		1.794 (1.169, 2.754)	0.007	1.793 (1.139, 2.823)	0.012
AFP, ng/mL (≥200 vs. <200)		1.483 (0.972, 2.264)	0.067	1.511 (0.951, 2.401)	0.081
Tumor diameter, cm (≥5 vs. <5)		2.203 (1.415, 3.431)	<0.001	2.262 (1.440, 3.554)	<0.001
Tumor number (Multiple vs. Single)		4.372 (2.146, 8.907)	<0.001	4.332 (2.022, 9.283)	<0.001
BCLC (B vs. 0+A)		0.732 (0.340, 1.578)	0.426	0.693 (0.302, 1.589)	0.386
Differentiation (Low vs. High and/or moderate)		2.944 (1.909, 4.541)	<0.001	2.997 (1.901, 4.725)	<0.001
MVI (Positive vs. Negative)		2.095 (1.198, 3.661)	<0.001	2.069 (1.130, 3.788)	0.018
NLR (≥2.4 vs. < 2.4)		1.294 (0.849, 1.972)	0.231	1.262 (0.805, 1.978)	0.311
Liver Cirrhosis (Positive vs. Negative)		0.800 (0.536, 1.194)	0.274	-	-

PSM, Propensity score matching; LR, liver resection; PA, postoperative adjuvant; TACE, transcatheter arterial chemoembolization; HAIC, hepatic artery perfusion chemotherapy; BCLC, barcelona clinic liver cancer; MVI, microvascular invasion; NLR, Neutrophil-Lymphocyte Ratio.

### 3.5 Sensitivity analyses

Building upon the primary analysis using PSM, we conducted sensitivity analyses employing IPTW. IPTW-adjusted Cox regression revealed that, compared to LR alone, the PA-TACE and PA-HAIC groups had significantly lower risks of recurrence (weighted HR 0.253, 95% CI 0.193–0.333; 0.157, 95% CI 0.114–0.216, respectively). Similarly, both groups exhibited significantly lower risks of mortality (weighted HR 0.341, 95% CI 0.202–0.488; 0.173, 95% CI 0.099–0.303, respectively). Weighted Kaplan–Meier curves further indicated clear separation between the groups, consistent with the direction of the PSM analysis (Supplementary Figure S1), thereby reinforcing the robustness of our conclusions.

### 3.6 Comparison of recurrence-free survival among patients stratified by combined recurrence risk factors

Based on high-risk factors for recurrence following radical resection of HCC, patients in the entire cohort were exploratorily stratified into subgroups. These included: 1) patients with a single recurrence risk factor, namely, the MVI-positive group, tumor diameter ≥5 cm group, multiple tumors group, and poorly differentiated tumor group; 2) patients with two recurrence risk factors, including MVI-positive + tumor diameter ≥5 cm (MVID group), MVI-positive + multiple tumors (MVIM group), and MVI-positive + poor differentiation (MVIP group); and 3) patients with three or more recurrence risk factors. The results showed no statistically significant differences in mRFS among patients with only one recurrence risk factor. Similarly, no significant differences in mRFS were observed among patients with two recurrence risk factors. However, mRFS decreased progressively with an increasing number of combined recurrence risk factors, and this trend was statistically significant (full details available in the Supplementary Table S4).

### 3.7 Comparison of recurrence-free survival among patients with different recurrence risk factors

In subgroup analyses stratified by recurrence risk factors, both PA-TACE and PA-HAIC were associated with significantly improved mRFS compared with LR. Notably, in subgroups defined by MVI-positive, multiple tumors or Diameter ≥5 cm, PA-HAIC conferred superior mRFS compared with PA-TACE. In contrast, among patients with poorly differentiated tumors or those with two or more combined recurrence risk factors, no significant difference in mRFS was observed between the PA-TACE and PA-HAIC groups (Table 5).

**TABLE 5:** Comparison of mRFS among patients with different recurrence risk factors undergoing different treatment strategies.

Group	mRFS, months			P value*		
	LR	PA-TACE	PA-HAIC	LR vs. PA-TACE	LR vs. PA-HAIC	PA-TACE vs. PA-HAIC
MVI-positive	38.00 (32.30, 43.70)	42.00 (38.87, 45.13)	51.00 (49.10, 52.90)	0.001	<0.001	0.007
Diameter ≥5 cm	22.00 (19.07, 24.94)	42.00 (36.19, 47.81)	52.00 (45.37, 58.63)	<0.001	<0.001	0.034
Multiple tumors	17.50 (13.34, 21.66)	45.00 (39.99, 50.01)	Not reached	<0.001	<0.001	0.040
Poor differentiation	15.00 (13.21, 16.79)	Not reached	43.00 (40.26, 45.74)	<0.001	<0.001	0.959
MVID	13.00 (11.70, 14.30)	31.00 (19.33, 42.67)	38.00 (33.21, 42.79)	<0.001	<0.001	0.399
MVIM	12.00 (8.80, 15.20)	37.00 (29.63, 44.37)	36.00 (29.71, 42.29)	<0.001	<0.001	0.972
MVIP	11.00 (9.17, 12.83)	35.00 (28.64, 41.36)	32.00 (30.59, 33.41)	<0.001	<0.001	0.902
Three or more recurrence risk factors	7.50 (6.59, 8.41)	18.50 (15.37, 21.63)	26.00 (20.47, 31.53)	<0.001	<0.001	0.211

mRFS, median recurrence-free survival time; LR, liver resection; PA, postoperative adjuvant; TACE, transcatheter arterial chemoembolization; HAIC, hepatic artery perfusion chemotherapy; MVI, microvascular invasion, MVID, MVI + tumor diameter ≥5 cm; MVIM, MVI + multiple tumors; MVIP, MVI + poor differentiation; *Comparison of RFS, among different adjuvant therapy regimens across subgroups.

### 3.8 Prognostic nomogram for recurrence-free survival

**FIGURE 4:**
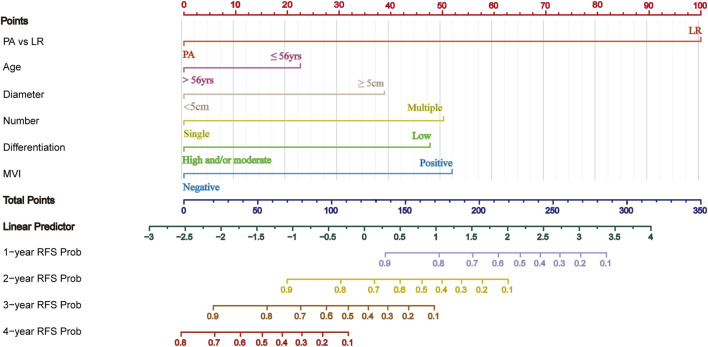
Nomogram for predicting RFS in HCC with HRRFs following radical resection. PA, Postoperative adjuvant; LR, liver resection; MVI, microvascular invasion; RFS, recurrence-free survival time.

Based on the results of multivariate Cox regression analysis for RFS, a predictive model was developed incorporating prognostic factors such as adjuvant therapy, age, tumor diameter, tumor number, tumor differentiation, and MVI. Patients from the First Affiliated Hospital of Chongqing Medical University (434 patients) were used as the training cohort, while patients from the Affiliated Yongchuan Hospital of Chongqing Medical University (175 patients) served as the validation cohort. The nomogram predicting RFS for the training cohort is shown in Figure 4. Each subtype of an independent risk factor was assigned a score. By summing the corresponding points and locating them on the total score scale, the predicted probability of RFS for each patient can be determined.

**FIGURE 5:**
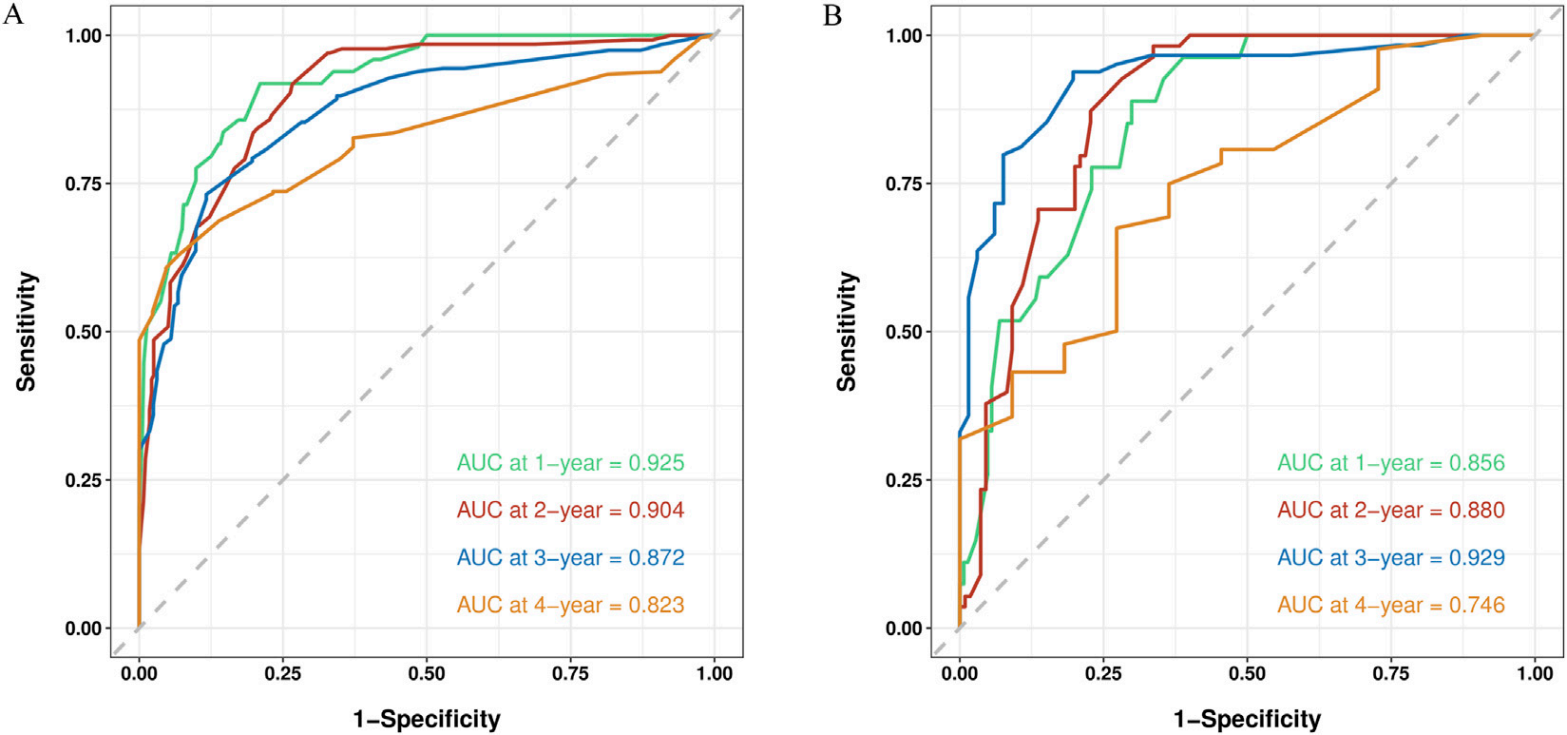
ROC curve of the nomogram in the training and validation cohort. **(A)** The AUC values for 1-, 2-, 3-, and 4-year RFS in the training cohort; **(B)** The AUC values for 1-, 2-, 3-, and 4-year RFS in the validation cohort.

The C-index and AUC are commonly used to assess the discriminative ability of the nomogram. A larger C-index indicates higher accuracy of the nomogram. In the training cohort, the C-index of the nomogram was 0.802 (95% CI 0.780, 0.824), and in the validation cohort, it was 0.799 (95% CI 0.764, 0.835). The ROC curve is another widely used tool for evaluating the discriminative ability of the nomogram. The area enclosed by the ROC curve and the horizontal and vertical axes is termed the AUC. In the training cohort, the AUC values for 1-, 2-, 3-, and 4-year RFS were 0.925, 0.904, 0.872, and 0.823, respectively. In the validation cohort, the corresponding AUC values were 0.856, 0.880, 0.929, and 0.746. Except for the 4-year RFS, the AUC values for the other time points were all >0.800, demonstrating a high consistency between the predicted and observed RFS probabilities (Figure 5). Furthermore, calibration plots for 1-, 2-, 3-, and 4-year RFS show good agreement between the predicted and actual observations (Supplementary Figure S2).

**FIGURE 6:**
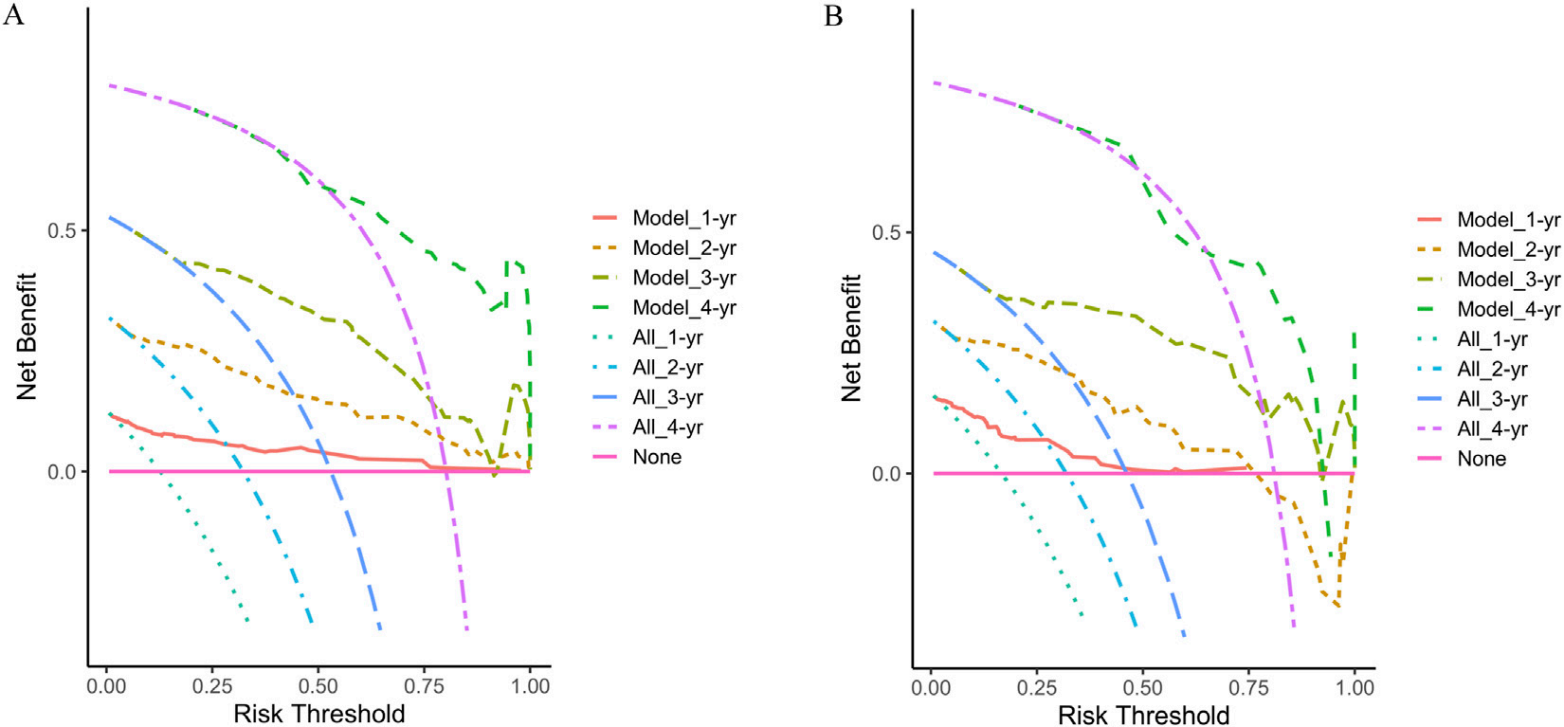
Decision curve analysis at 1-year, 2-year, 3-year, and 4-year of the two cohorts. **(A)** The training cohort; **(B)** The validation cohort.

Finally, the clinical utility of the nomogram was assessed using decision curve analysis (DCA). The horizontal axis of the curve represents the threshold probability, while the vertical axis represents net benefit. When no patients experience recurrence and no clinical intervention is applied, the net benefit is represented by the ‘None’ line. Conversely, when all patients experience recurrence and undergo clinical intervention, the net benefit is represented by the ‘All’ line. If the net benefit rate corresponding to the threshold probability lies to the right of both the ‘All’ and ‘None’ lines (with the net benefit rates for each threshold probability connected to form a red line), this indicates that the predictive model has good clinical utility. In our study, both in the training and validation cohorts, the 1-year, 2-year, 3-year, and 4-year DCA curves demonstrated that within a certain range of threshold probabilities, the probability of patients benefiting from the nomogram was greater than that of the two extreme scenarios, with a net benefit greater than zero. This suggests that the model has high clinical utility (Figure 6).

### 3.9 Adverse events and subsequent anti-tumor therapy in the PA-TACE and PA-HAIC groups

All adverse events (AEs) related to PA-TACE and PA-HAIC were mild and managed symptomatically during hospitalization. In the Entire cohort, the most common complications are summarized as follows: elevated transaminases and pain occurred more frequently in the PA-TACE group (33.82% vs. 24.49%; 28.68% vs. 19.05%, respectively), whereas nausea/vomiting were more frequent in the PA-HAIC group (22.43% vs. 37.41%). The incidence of other complications was generally similar between the two groups, and there were no statistically significant differences in grade 3 AE. Details are summarized in Supplementary Table S5.

Patients in both groups received subsequent anti-tumor therapies after recurrence, including curative treatment, TACE, HAIC, immunotherapy and targeted therapy. A greater proportion of patients with recurrence in the PA-HAIC group underwent curative treatment, whereas those in the PA-TACE group had relatively fewer opportunities to receive such treatment (Supplementary Table S6).

## 4 Discussion

In our study, we investigated the efficacy of PA-TACE and PA-HAIC in comparison to liver resection alone in patients with high-risk recurrence after radical resection for HCC. The results demonstrated that patients receiving PA-HAIC or PA-TACE had better survival outcomes, including improved RFS and OS, compared to those undergoing radical resection alone. Additionally, multivariate Cox regression analysis confirmed that PA-TACE and PA-HAIC were independent protective factors for survival outcomes, consistent with previous studies (Yang et al., 2021; Xiang et al., 2024; Hu et al., 2023). Additionally, two studies involving HCC patients from multiple centers across China ultimately demonstrated that younger patients, compared to older ones, exhibit greater tumor invasiveness and metastatic potential, leading to higher postoperative recurrence rates and tumor-specific mortality. This finding is consistent with the conclusion from our study’s multivariate Cox analysis, which identified age ≤56 years as an independent risk factor for both RFS and OS (Diao et al., 2021; Pu et al., 2022).

Compared with PA-TACE, PA-HAIC significantly improved mRFS and 1-, 2-, and 4-year RFS, in line with the findings of Wen et al. (2024). These advantages may stem from differences in drug concentration, delivery method, and the degree of ischemic liver injury between TACE and HAIC. TACE targets tumor cells released during surgical compression and microscopic lesions undetectable by imaging or intraoperative assessment by delivering localized chemotherapy with embolic agents (Chen et al., 2013). Lipiodol deposited in the tumor vasculature reduces intratumoral blood flow, depriving the tumor of oxygen and nutrients while prolonging local drug retention, thereby enhancing chemotherapeutic exposure (Gaba et al., 2018). However, vascular embolization induced by TACE leads to ischemic liver injury, upregulating the expression of hypoxia-inducible factor-1α, vascular endothelial growth factor, and epithelial-mesenchymal transition (EMT) regulators. This cascade activates multiple downstream signaling pathways, including the Wnt/β-catenin pathway. These processes promote facilitate peritumoral hepatic fibrosis, exacerbate hypoxia, and enhance immunosuppressive effects within the tumor microenvironment (Qu et al., 2015; Ader et al., 2008; Fang et al., 2013; Xu et al., 2019; Lin et al., 2021). Additionally, TACE-induced alterations in the tumor vasculature, such as tortuous vessels, uneven blood flow distribution, leakage, and elevated interstitial pressure—impede the effective delivery of chemotherapy agents to tumor regions, thereby limiting the therapeutic efficacy of TACE (Albini and Sporn, 2007). Collectively, these factors contribute to tumor recurrence, progression, and metastasis. Mechanistically, HAIC capitalizes on two key pharmacokinetic advantages: first-pass hepatic extraction and enhanced tumor penetration. Studies have demonstrated that HAIC of floxuridine or 5-fluorouracil can achieve intrahepatic uptake rates of up to 90% and 19%–90%, respectively. These rates significantly exceed those observed with conventional intravenous administration. Accordingly, intratumoral drug concentrations are also markedly elevated. In addition, sustained high-flow infusion creates increased interstitial pressure gradients, improving intratumoral drug distribution. These synergistic mechanisms augment the efficacy of chemotherapy while mitigating extrahepatic toxicity (Leal and Kingham, 2015; Liang et al., 2013; Ganeshan et al., 2008). A study suggests that HAIC exerts immunomodulatory effects in HCC. It is hypothesized that HAIC induces the upregulation of major histocompatibility complex class I molecules on tumor cells. These tumor cells are then processed and presented by dendritic cells and B cells, initiating antigen-specific T cell responses. This process promotes the differentiation of naïve CD4^+^ T cells into T follicular helper cells and T helper cells, thereby modulating the immunosuppressive tumor microenvironment in HCC. These effects collectively contribute to enhancing anti-tumor immunity (Huang et al., 2025). Additionally, HAIC does not embolize the tumor’s blood supply, thereby avoiding hypoxia-induced tumor suppression. This approach effectively alleviates and reduces liver fibrosis, tumor recurrence, and metastasis (Zhu et al., 2024). Furthermore, A meta-analysis has shown that for patients with unresectable HCC, the incidence of severe adverse events (death, progressive liver dysfunction, or liver failure) induced by HAIC is significantly lower than that of TACE, providing evidence that HAIC causes less liver damage than TACE (Liu et al., 2022).

In our study, patients receiving PA-HAIC showed improved OS compared with those receiving PA-TACE. This may be explained by two factors: first, PA-TACE adversely affects the tumor microenvironment more than PA-HAIC, potentially promoting tumor recurrence and reducing sensitivity to subsequent anti-tumor therapies, resulting in poorer outcomes; second, patients in the PA-HAIC group had relatively more opportunities to undergo curative treatment during follow-up, indicating that they had a relatively lower tumor burden at recurrence, which contributed to their improved prognosis. Nonetheless, the relatively small number of patients reaching the endpoint and limited follow-up may have influenced these results. Therefore, extending the follow-up period is highly necessary.

Subgroup analyses further revealed that the risk of recurrence increased with the number of high-risk factors. Consequently, more aggressive postoperative adjuvant therapies should be considered as the number of risk factors increases. Moreover, our study demonstrated that among patients with only MVI positivity, multiple tumors, or tumor diameter ≥5 cm, PA-HAIC conferred greater benefit in improving recurrence-free survival compared to PA-TACE. However, in patients with two or more high-risk recurrence factors, no significant difference in RFS improvement was observed between PA-HAIC and PA-TACE. This also demonstrates that PA-TACE exhibits both tumor-suppressive and tumor-promoting effects. In patients with only one high-risk recurrence factor, the gap between the tumor-suppressive and tumor-promoting effects of PA-TACE was smaller, leading to a lower benefit for patients, thus making PA-HAIC more beneficial than PA-TACE. As the number of high-risk recurrence factors increased, the difference between the tumor-suppressive and tumor-promoting effects of PA-TACE gradually widened, resulting in a progressively significant therapeutic effect of PA-TACE. Subgroup analyses from multiple studies have shown that PA-TACE is more beneficial for hepatocellular carcinoma patients with two or more recurrence risk factors after curative resection than for those with only one recurrence risk factor, thus providing partial support for our hypothesis and conclusions (Wang et al., 2019; Wang et al., 2020; Qi et al., 2019). Additionally, MVI remains one of the most significant high-risk factors for HCC, consistent with prior evidence. However, broader high-risk classifications should be considered exploratory and hypothesis-generating, necessitating external validation. Notably, tumor size and multiplicity are strongly associated with MVI, indicating collinearity between these factors (Zhang et al., 2018). Consequently, these variables are not entirely independent, which may limit the interpretability of subgroup analyses. Therefore, results should be interpreted with caution.

This study still has several limitations. As a retrospective study, inherent biases are unavoidable. We employed a multicenter design, established strict inclusion and exclusion criteria, and utilized PSM and subgroup analyses. However, the limited sample size, particularly among patients receiving PA-HAIC, may still introduce bias in the results. Further expansion of the sample size is needed. Second, different aetiologies of HCC may influence responses to PA-TACE and PA-HAIC. However, our cohort was dominated by patients with HBV infection, while cases of HCV and alcoholic hepatitis were relatively few, which limits the generalisability of our findings. Lastly, this study predominantly enrolled HCC patients from China, and the findings are therefore applicable only to Chinese or broader Asian populations, and should not be extrapolated to patients globally.

## 5 Conclusion

In HCC patients with high-risk recurrence following radical resection, PA-HAIC significantly improves RFS compared to PA-TACE, but only in patients with MVI, tumor diameter ≥5 cm, or multiple tumors. No statistically significant difference in efficacy was observed between the two treatments in patients with poorly differentiated tumors, two or more recurrence risk factors.

## Data Availability

The raw data supporting the conclusions of this article will be made available by the authors, without undue reservation.
