# Supplementary material for: Comparative efficacy of postoperative adjuvant transcatheter arterial chemoembolization and hepatic artery infusion chemotherapy in patients with BCLC stage 0-B hepatocellular carcinoma at high risk of recurrence following radical resection
Source: Front Pharmacol. 2025 Sep 19;16:1657794. doi: 10.3389/fphar.2025.1657794 (PMC12492446; doi:10.3389/fphar.2025.1657794)
Supplement: Supplementary file 1 [file Supplementaryfile1.docx]

***Comparative Efficacy of Postoperative Adjuvant Transhepatic Arterial Chemoembolization and Hepatic Artery Infusion Chemotherapy in Patients with BCLC Stage 0-B Hepatocellular Carcinoma at High Risk of Recurrence Following Radical Resection***

Xu Feng^1,2^, Yupei Ao^3^, Jiarui Liu^1^, Xuanjun Liu^1^, Zheng-Rong Shi^2^ and Chengjia Tang^1*^

^1^Department of Hepatobiliary Surgery, The First Affiliated Hospital of Chongqing Medical University, (China), Chongqing

^2^ Department of Hepatobiliary Surgery, The Affiliated Yongchuan Hospital of Chongqing Medical University

^3^ Health Screening Centre, Chongqing Western Hospital, (China), Chongqing

^*^Correspondence should be addressed to Chengjia Tang; 3096275702@qq.com

Supplementary table 1 Comparison of RFS in HCC patients receiving different postoperative adjuvant therapy

| Characteristics | | mRFS (months) | 1-year RFS rate (%) | 2-year RFS rate (%) | 3-year RFS rate (%) | 4-year RFS rate (%) |
| --- | --- | --- | --- | --- | --- | --- |
| the Entire cohort | LR | 16.00 (14.20-17.80) | 67.80 (61.14-74.46) | 67.80 (61.14-74.46) | 15.10 (9.02-21.18) | - |
|  | PA-TACE | 38.50 (36.81-40.19) | 92.30 (89.16-95.44) | 81.30 (76.60-86.00) | 62.30 (56.22-68.38) | 22.00 (15.73-28.72) |
|  | PA-HAIC | 42.00 (38.21-45.79) | 98.60 (96.64-100.00) | 90.10 (85.20-95.00) | 65.40 (57.36-73.44) | 32.50 (23.48-41.52) |
| the PSM cohort | LR | 16.50 (14.26–18.74) | 67.30 (60.24-74.36) | 32.70 (25.64-39.76) | 15.30 (8.83-21.77) | - |
|  | PA-TACE | 39.00 (37.23–40.77) | 92.20 (88.87-95.53) | 81.90 (77.20-86.60) | 63.00 (56.73-69.27) | 21.30 (15.03-27.57) |
|  | PA-HAIC | 46.00 (42.20–49.80) | 100.00 (100.00-100.00) | 94.90 (90.98-98.82) | 94.90 (90.98-98.82) | 36.40 (26.21-46.59) |
| Comparison | | p value | | | | |
| the Entire cohort | LR vs PA-TACE | <0.001 | <0.001 | <0.001 | <0.001 | - |
|  | LR vs PA-HAIC | <0.001 | <0.001 | <0.001 | <0.001 | - |
|  | PA-TACE vs PA-HAIC | 0.008 | 0.003 | 0.024 | 0.620 | 0.018 |
| the PSM cohort | LR vs PA-TACE | <0.001 | <0.001 | <0.001 | <0.001 | - |
|  | LR vs PA-HAIC | <0.001 | <0.001 | <0.001 | <0.001 | - |
|  | PA-TACE vs PA-HAIC | <0.001 | <0.001 | <0.001 | 0.113 | 0.002 |

PSM, Propensity score matching; LR, liver resection; PA, Postoperative adjuvant; TACE, transcatheter arterial chemoembolization; HAIC, Hepatic artery perfusion chemotherapy; mRFS, median recurrence-free survival time.

Supplementary table 2 Comparison of OS in HCC patients receiving different postoperative adjuvant therapy

| Characteristics | | mOS (months) | 2-year OS rate (%) | 3-year OS rate (%) | 4-year OS rate (%) | 5-year OS rate (%) |
| --- | --- | --- | --- | --- | --- | --- |
| the Entire cohort | LR | 52.0 (49.26–54.75) | 96.70 (94.15–99.25) | 92.40 (88.28–96.52) | 60.40 (48.84–71.96) | 34.50 (19.01–49.98) |
|  | PA-TACE | 68.0 (62.02–73.98) | 99.20 (98.22–100.00) | 96.60 (94.25–98.95) | 85.60 (80.50–90.70) | 73.30 (64.28–82.32) |
|  | PA-HAIC | Not reached | 100.00 (100.00–100.00) | 98.50 (96.34–100.00) | 92.00 (86.90–97.10) | 73.70 (61.55–85.85) |
| the PSM cohort | LR | 54.0 (48.33–59.67) | 67.30 (60.24-74.36) | 32.70 (25.64-39.76) | 15.30 (8.83-21.77) | - |
|  | PA-TACE | 68.0 (58.13–77.89) | 92.20 (88.87-95.53) | 81.90 (77.20-86.60) | 63.00 (56.73-69.27) | 21.30 (15.03-27.57) |
|  | PA-HAIC | Not reached | 100.00 (100.00-100.00) | 94.90 (90.98-98.82) | 94.90 (90.98-98.82) | 36.40 (26.21-46.59) |
| Comparison | | p value | | | | |
| the Entire cohort | LR vs PA-TACE | <0.001 | 0.069 | 0.048 | <0.001 | <0.001 |
|  | LR vs PA-HAIC | <0.001 | 0.038 | 0.010 | <0.001 | <0.001 |
|  | PA-TACE vs PA-HAIC | 0.155 | 0.543 | 0.342 | 0.065 | 0.946 |
| the PSM cohort | LR vs PA-TACE | <0.001 | 0.066 | 0.027 | <0.001 | <0.001 |
|  | LR vs PA-HAIC | <0.001 | 0.085 | 0.005 | <0.001 | <0.001 |
|  | PA-TACE vs PA-HAIC | 0.043 | 1.000 | 0.282 | 0.008 | 0.245 |

PSM, Propensity score matching; LR, liver resection; PA, Postoperative adjuvant; TACE, transcatheter arterial chemoembolization; HAIC, Hepatic artery perfusion chemotherapy; mOS, median Overall survival time.

Supplementary table 3.A Univariate COX Regression Analysis of RFS in the Entire Cohort and PSM Cohort

| Characteristics | | the Entire cohort | | the PSM cohort | |
| --- | --- | --- | --- | --- | --- |
|  |  | HR (95% CI) | p | HR (95% CI) | p |
| Type of treatment | LR | Reference | - | Reference | - |
|  | PA-TACE | 0.247 (0.196, 0.312) | <0.001 | 0.245 (0.193, 0.313) | <0.001 |
|  | PA-HAIC | 0.181 (0.137, 0.239) | <0.001 | 0.156 (0.115, 0.212) | <0.001 |
| Age, yr (≤56 vs >56) | | 1.622 (1.343, 1.959) | <0.001 | 1.592 (1.306, 1.940) | <0.001 |
| Gender (Female vs male) | | 0.882 (0.676, 1.152) | 0.882 | 0.907 (0.683, 1.206) | 0.503 |
| Hepatitis | No hepatitis | Reference | - | Reference | - |
|  | HBV | 1.117 (0.756, 1.652) | 0.578 | 1.111 (0.745, 1.656) | 0.606 |
|  | HCV | 0.841 (0.488, 1.449) | 0.532 | 0.873 (0.495, 1.541) | 0.640 |
|  | AH | 0.947 (0.526, 1.704) | 0.855 | 1.083 (0.587, 1.998) | 0.798 |
| Liver Cirrhosis (Positive vs Negative) | | 0.954 (0.789, 1.152) | 0.622 | 0.985 (0.785, 1.169) | 0.672 |
| AFP, ng/ml (≥ 200 vs < 200) | | 1.393 (1.136, 1.708) | 0.001 | 1.389 (1.118, 1.725) | 0.003 |
| Tumor diameter, cm (≥5 vs < 5) | | 1.585 (1.311, 1.916) | <0.001 | 1.640 (1.342, 2.005) | <0.001 |
| Tumor number (Multiple vs Single) | | 1.465 (1.190, 1.803) | <0.001 | 1.318 (1.052, 1.652) | 0.016 |
| BCLC grade (B vs 0+A) | | 1.381 (1.091, 1.747) | 0.007 | 1.307 (1.013, 1.688) | 0.039 |
| Child-pugh score (6 vs 5) | | 1.316 (1.024, 1.690) | 0.032 | 1.314 (1.000, 1.727) | 0.050 |
| Hemoglobin, g  /L, (≤140 vs > 140) | | 1.205 (0.999, 1.454) | 0.051 | 1.213 (0.995, 1.477) | 0.056 |
| NLR (≥ 2.4 vs < 2.4) | | 1.206 (1.000, 1.455) | 0.050 | 1.175 (0.965, 1.431) | 0.108 |
| PLR (≥ 145 vs < 145) | | 0.998 (0.814, 1.225) | 0.988 | 0.976 (0.787, 1.210) | 0.824 |
| ALBI grade (2 vs 1) | | 1.177 (0.968, 1.432) | 0.103 | 1.161 (0.942, 1.431) | 0.161 |
| PALBI grade | 1 | Reference |  | Reference |  |
|  | 2 | 1.111 (0.911, 1.356) | 0.300 | 1.090 (0.882, 1.347) | 0.424 |
|  | 3 | 1.455 (0.832, 2.541) | 0.188 | 1.007 (0.497, 2.039) | 0.985 |
| Total protein (< 70 vs ≥ 70) | | 1.025 (0.850, 1.235) | 0.797 | 1.024 (0.841, 1.247) | 0.812 |
| ALT, U/L (≥ 34 vs < 34) | | 1.045 (0.867, 1.260) | 0.645 | 1.073 (0.881, 1.306) | 0.483 |
| AST, U/L (≥ 34 vs < 34) | | 1.101 (0.913, 1.327) | 0.315 | 1.129 (0.927, 1.374) | 0.228 |
| ALP, U/L (≥ 90 vs < 90) | | 1.189 (0.986, 1.434) | 0.069 | 1.178 (0.968, 1.435) | 0.102 |
| Differentiation (Low vs High and/or moderate） | | 1.516 (1.241, 1.852) | <0.001 | 1.504 (1.220, 1.853) | <0.001 |
| MVI (Positive vs Negative) | | 1.675 (1.351, 2.077) | <0.001 | 1.830 (1.454, 2.302) | <0.001 |
| Resection pattern (Nonanatomic vs Anatomic) | | 1.129 (0.902, 1.415) | 0.290 | 1.131 (0.890, 1.437) | 0.314 |
| Blood transfusion (Yes vs No) | | 1.258 (0.945, 1.658) | 0.103 | 1.289 (0.963, 1.726) | 0.088 |

PSM, Propensity score-match; PA, Postoperative adjuvant; TACE, transcatheter arterial chemoembolization; HAIC, Hepatic artery perfusion chemotherapy; HBV, Hepatitis B virus; HCV, Hepatitis C virus; AH, alcoholic hepatitis; AFP, Alpha-fetoprotein; BCLC, Barcelona Clinic Liver Cancer; NLR, Neutrophil-Lymphocyte Ratio; PLR, Platelet-Lymphocyte Ratio, ALBl = (log10 total bilirubin*0.66) + (albumin * -0.085), ALBI grade 1, ≤ -2.60; ALBI grade 2, -2.60 ~ -1.39; PALBI = (2.02*log10 total bilirubin)-0.37*(log10 total bilirubin)^2^-(0.04*albumin) - (3.48*log10 platelet) + 1.01*(log10 platelet)^2^, PALBI grade 1, ≤ -2.53; ALBI grade 2, -2.53 ~ -2.09; PALBI grade 3, > -2.09; ALT, alanine aminotransferase; AST, Alanine aminotransferase; ALP, Alkaline phosphatase; MVI, microvascular invasion.

Supplementary table 3.B Univariate COX Regression Analysis of OS in the Entire Cohort and PSM Cohort.

| Characteristics | | the Entire cohort | | the PSM cohort | |
| --- | --- | --- | --- | --- | --- |
|  |  | HR (95% CI) | p | HR (95% CI) | p |
| Type of treatment | LR | Reference |  | Reference |  |
|  | PA-TACE | 0.305 (0.200, 0.464) | <0.001 | 0.297 (0.192, 0.461) | <0.001 |
|  | PA-HAIC | 0.211 (0.124, 0.359) | <0.001 | 0.155 (0.083, 0.290) | <0.001 |
| Age, yr (≤56 vs >56) | | 2.696 (1.7989, 4.044) | <0.001 | 2.795 (1.806, 4.326) | <0.001 |
| Gender (Male vs Female) | | 1.041 (0.612, 1.770) | 0.882 | 1.124 (0.626, 2.019) | 0.691 |
| Hepatitis | No hepatitis | Reference | - | Reference | - |
|  | HBV | 0.862 (0.434, 1.714) | 0.862 | 0.892 (0.430, 1.849) | 0.758 |
|  | HCV | 0.761 (0.283, 2.047) | 0.588 | 0.733 (0.240, 2.242) | 0.586 |
|  | AH | 0.842 (0.299, 2.366) | 0.744 | 1.070 (0.371, 3.087) | 0.900 |
| Liver Cirrhosis (Positive vs Negative) | | 0.674 (0.463, 0.980) | 0.039 | 0.697 (0.467, 1.040) | 0.077 |
| AFP, ng/ml (≥ 200 vs < 200) | | 2.429 (1.668, 3.537) | <0.001 | 2.407 (1.607, 3.608) | <0.001 |
| Tumor diameter, cm (≥5 vs ＜5) | | 2.176 (1.493, 3.173) | <0.001 | 2.216 (1.482, 3.313) | <0.001 |
| Tumor number (Multiple vs Single) | | 2.116 (1.434, 3.122) | <0.001 | 1.892 (1.235, 2.898) | 0.003 |
| BCLC grade (B vs 0+A) | | 2.103 (1.380, 3.205) | <0.001 | 1.899 (1.186, 3.040) | 0.008 |
| Child-pugh score (6 vs 5) | | 1.018 (0.598, 1.734) | 0.948 | 0.922 (0.502, 1.691) | 0.792 |
| Hemoglobin, g  /L (≤140 vs > 140) | | 1.097 (0.755, 1.595) | 0.626 | 1.140 (0.764, 1.701) | 0.522 |
| NLR (≥ 2.4 vs < 2.4) | | 1.828 (1.218, 2.744) | 0.004 | 1.791 (1.160, 2.765) | 0.009 |
| PLR (≥ 145 vs < 145) | | 1.279 (0.861, 1.902) | 0.223 | 1.253 (0.819, 1.913) | 0.299 |
| ALBI grade (2 vs 1) | | 1.015 (0.678, 1.519) | 0.942 | 0.925 (0.591, 1.448) | 0.734 |
| PALBI grade | 1 | Reference | - | Reference | - |
|  | 2 | 1.034 (0.689, 1.553) | 0.870 | 0.959 (0.616, 1.492) | 0.852 |
|  | 3 | 2.351 (0.852, 6.488) | 0.099 | 2.405 (0.750, 7.711) | 0.140 |
| Total protein, g/L (< 70 vs ≥ 70) | | 1.146 (0.788, 1.668) | 0.476 | 1.177 (0.788, 1.759) | 0.425 |
| ALT, U/L (≥ 34 vs < 34) | | 0.768 (0.527, 1.118) | 0.168 | 0.754 (0.505, 1.128) | 0.170 |
| AST, U/L (≥ 34 vs < 34) | | 0.929 (0.638, 1.352) | 0.700 | 1.017 (0.680, 1.520) | 0.934 |
| ALP, U/L (≥ 90 vs < 90) | | 1.105 (0.759, 1.608) | 0.603 | 1.134 (0.758, 1.696) | 0.540 |
| Differentiation (Low vs High and/or moderate） | | 2.190 (1.499, 3.198) | <0.001 | 2.125 (1.418, 3.187) | <0.001 |
| MVI (Positive vs Negative) | | 2.125 (1.279, 3.532) | 0.004 | 2.347 (1.348, 4.088) | 0.003 |
| Resection pattern (Nonanatomic vs Anatomic) | | 1.123 (0.714, 1.767) | 0.616 | 1.133 (0.698, 1.839) | 0.613 |
| Blood transfusion (Yes vs No) | | 0.822 (0.450, 1.501) | 0.524 | 0.816 (0.423, 1.575) | 0.545 |

PSM, Propensity score-match; PA, Postoperative adjuvant; TACE, transcatheter arterial chemoembolization; HAIC, Hepatic artery perfusion chemotherapy; HBV, Hepatitis B virus; HCV, Hepatitis C virus; AH, alcoholic hepatitis; AFP, Alpha-fetoprotein; BCLC, Barcelona Clinic Liver Cancer; NLR, Neutrophil-Lymphocyte Ratio; PLR, Platelet-Lymphocyte Ratio, ALBl = (log10 total bilirubin*0.66) + (albumin * -0.085), ALBI grade 1, ≤ -2.60; ALBI grade 2, -2.60 ~ -1.39; PALBI = (2.02*log10 total bilirubin)-0.37*(log10 total bilirubin)^2^-(0.04*albumin) - (3.48*log10 platelet) + 1.01*(log10 platelet)^2^, PALBI grade 1, ≤ -2.53; ALBI grade 2, -2.53 ~ -2.09; PALBI grade 3, > -2.09; ALT, alanine aminotransferase; AST, Alanine aminotransferase; ALP, Alkaline phosphatase; MVI, microvascular invasion.

Supplementary table 4. Comparison of mRFS in HCC patients with different recurrence risk factors

| Group | mRFS, months | A single recurrence risk factor | | | | Two recurrence risk factors | | | Three or more recurrence risk factors |
| --- | --- | --- | --- | --- | --- | --- | --- | --- | --- |
|  |  | MVI-positive | Diameter ≥5 cm | Multiple tumors | Poor differentiation | MVID | MVIM | MVIP |  |
| MVI-positive | 42.00 (39.39, 44.61) | - | 0.624* | 0.081 | 0.068 | <0.001 | <0.001 | <0.001 | <0.001 |
| Diameter ≥5 cm | 43.00 (37.03, 48.97) | 0.624 | - | 0.285 | 0.392 | <0.001 | <0.001 | <0.001 | <0.001 |
| Multiple tumors | 40.00 (35.80, 44.20) | 0.081 | 0.285 | - | 0.966 | <0.001 | <0.001 | 0.005 | <0.001 |
| Poor differentiation | 41.50 (37.82, 45.18) | 0.068 | 0.392 | 0.966 | - | 0.002 | <0.001 | 0.012 | <0.001 |
| MVID | 26.00 (21.26, 30.75) | <0.001 | <0.001 | <0.001 | 0.002 | - | 0.456 | 0.584 | <0.001 |
| MVIM | 26.00 (15.28, 36.72) | <0.001 | <0.001 | <0.001 | <0.001 | 0.456 | - | 0.303 | <0.001 |
| MVIP | 29.00 (22.99, 35.01) | <0.001 | <0.001 | 0.005 | 0.012 | 0.584 | 0.303 | - | <0.001 |
| Three or more recurrence risk factors | 18.00 (15.21, 20.79) | <0.001 | <0.001 | <0.001 | <0.001 | <0.001 | <0.001 | <0.001 | - |

mRFS, median recurrence-free survival time; MVI, microvascular invasion, MVID, MVI-positive + tumor diameter ≥5 cm; MVIM, MVI-positive + multiple tumors; MVIP, MVI-positive + poor differentiation.

* P-value for the comparison of RFS among patient groups stratified by different risk factors.

Supplementary table 5.A Comparison of adverse events occurring in patients with HCC who

received PA-TACE and PA-HAIC in the Entire cohort

| Adverse events | Grade 1-2, n (%) | | | Grade 3, n (%) | | |
| --- | --- | --- | --- | --- | --- | --- |
|  | PA-TACE  (n=272) | PA-HAIC  (n=147) | p | PA-TACE  (n=272) | PA-HAIC  (n=147) | p |
| Elevated AST/ALT | 98 (36.03) | 36 (24.49) | 0.016 | 21 (7.72) | 10 (6.80) | 0.732 |
| Pain | 78 (28.68) | 28 (19.05) | 0.030 | 9 (3.31) | 6 (4.08) | 0.684 |
| Nausea/Vomiting | 61 (22.43) | 55 (37.41) | 0.001 | 0 (0.00) | 0 (0.00) | - |
| Fever | 57 (20.96) | 38 (25.85) | 0.254 | 12 (4.41) | 6 (4.08) | 0.874 |
| Neutropenia | 51 (18.75) | 19 (12.93) | 0.127 | 10 (3.68) | 5 (3.40) | 0.885 |
| Elevated total bilirubin | 41 (15.07) | 21 (14.29) | 0.828 | 18 (6.61) | 6 (4.08) | 0.286 |
| Thrombocytopenia | 23 (8.46) | 18 (12.24) | 0.213 | 6 (2.21) | 3 (2.04) | 0.919 |
| Fatigue | 14 (5.15) | 8 (5.44) | 0.897 | 0 (0.00) | 0 (0.00) | - |

PA, Postoperative adjuvant; TACE, transcatheter arterial chemoembolization; HAIC, Hepatic artery perfusion chemotherapy; ALT, alanine aminotransferase; AST, Alanine aminotransferase.

Supplementary table 5.B Comparison of adverse events occurring in patients with HCC who

received PA-TACE and PA-HAIC in the PSM cohort

| Adverse events | Grade 1-2, n (%) | | | Grade 3, n (%) | | |
| --- | --- | --- | --- | --- | --- | --- |
|  | PA-TACE  (n=257) | PA-HAIC  (n=122) | p | PA-TACE  (n=257) | PA-HAIC  (n=122) | p |
| Elevated AST/ALT | 92 (35.78) | 31 (25.41) | 0.044 | 18 (7.00) | 9 (7.38) | 0.163 |
| Pain | 75 (29.18) | 24 (19.67) | 0.049 | 9 (3.50) | 5 (4.10) | 0.774 |
| Nausea/Vomiting | 57 (22.17) | 50 (40.98) | <0.001 | 0 (0.00) | 0 (0.00) | - |
| Fever | 55 (21.40) | 34 (27.87) | 0.165 | 12 (4.67) | 6 (4.92) | 0.915 |
| Neutropenia | 49 (19.07) | 18 (14.75) | 0.304 | 10 (3.89) | 5 (4.10) | 0.923 |
| Elevated total bilirubin | 39 (15.18) | 19 (15.57) | 0.920 | 16 (6.23) | 5 (4.10) | 0.398 |
| Thrombocytopenia | 23 (8.95) | 16 (13.11) | 0.410 | 6 (2.33) | 3 (2.46) | 0.941 |
| Fatigue | 13 (5.06) | 8 (6.56) | 0.386 | 0 (0.00) | 0 (0.00) | - |

PA, Postoperative adjuvant; TACE, transcatheter arterial chemoembolization; HAIC, Hepatic artery perfusion chemotherapy; ALT, alanine aminotransferase; AST, Alanine aminotransferase.

Supplementary table 6 Post-recurrence treatment strategies in the PA-TACE and PA-HAIC groups

| Anti-tumour therapy | the Entire cohort | | | the PSM cohort | | |
| --- | --- | --- | --- | --- | --- | --- |
|  | PA-TACE (n=191) | PA-HAIC (n=96) | p | PA-TACE (n=181) | PA-HAIC (n=78) | p |
| Curative treatment^1^+ Comprehensive treatment^2^ | 54 (28.27) | 38 (39.58) | 0.053 | 51 (28.18) | 33 (42.31) | 0.026 |
| Comprehensive treatment | 80 (41.88) | 34 (35.42) | 0.291 | 79 (43.65) | 26 (33.33) | 0.121 |
| TACE/HAIC+ Immune/targeted therapy | 24 (12.57) | 8 (8.33) | 0.282 | 22 (12.15) | 7 (8.97) | 0.456 |
| Immune-targeted therapy only | 13 (6.81) | 6 (6.25) | 0.858 | 12 (6.630 | 5 (6.41) | 0.948 |
| TACE/HAIC only | 11 (5.76) | 6 (6.25) | 0.868 | 11 (6.08) | 4 (5.13) | 0.762 |
| Palliative care | 9 (4.71) | 4 (4.17) | 0.834 | 6 (3.31) | 3 (3.85) | 0.830 |

PA, Postoperative adjuvant; TACE, transcatheter arterial chemoembolization; HAIC, Hepatic artery perfusion chemotherapy; ALT, alanine aminotransferase; AST, Alanine aminotransferase.

^1^ Curative treatment includes liver resection, liver transplantation, and radiofrequency ablation;

^2^ Comprehensive treatment refers to TACE/HAIC combined with targeted therapy and immunotherapy;

^3^The immunotherapy treatment regimen mainly includes Tislelizumab, Camrelizumab, or Atezolizumab; The targeted therapy treatment regimen mainly includes Sorafenib, Lenvatinib, Regorafenib, Apatinib, or Bevacizumab.

Supplementary figure 1 IPTW-Adjusted Kaplan–Meier Curve


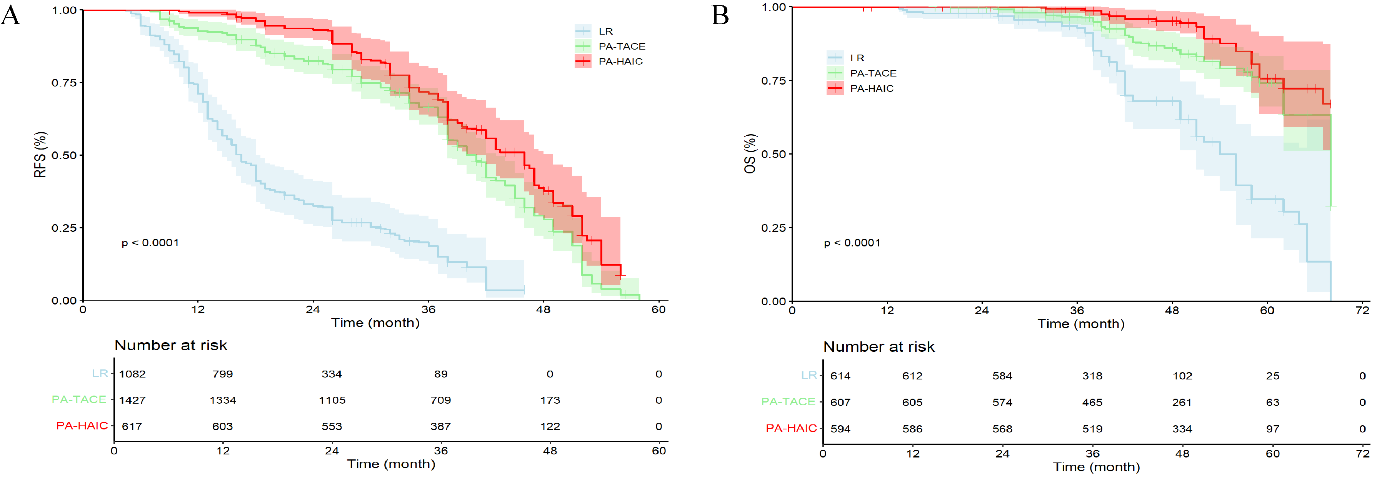


A, the recurrence-free survival; B, the overall survival; LR, liver resection; PA, Postoperative adjuvant; TACE, transcatheter arterial chemoembolization; HAIC, Hepatic artery perfusion chemotherapy.

Supplementary figure 2 Calibration curve of the nomogram in the training and validation cohort


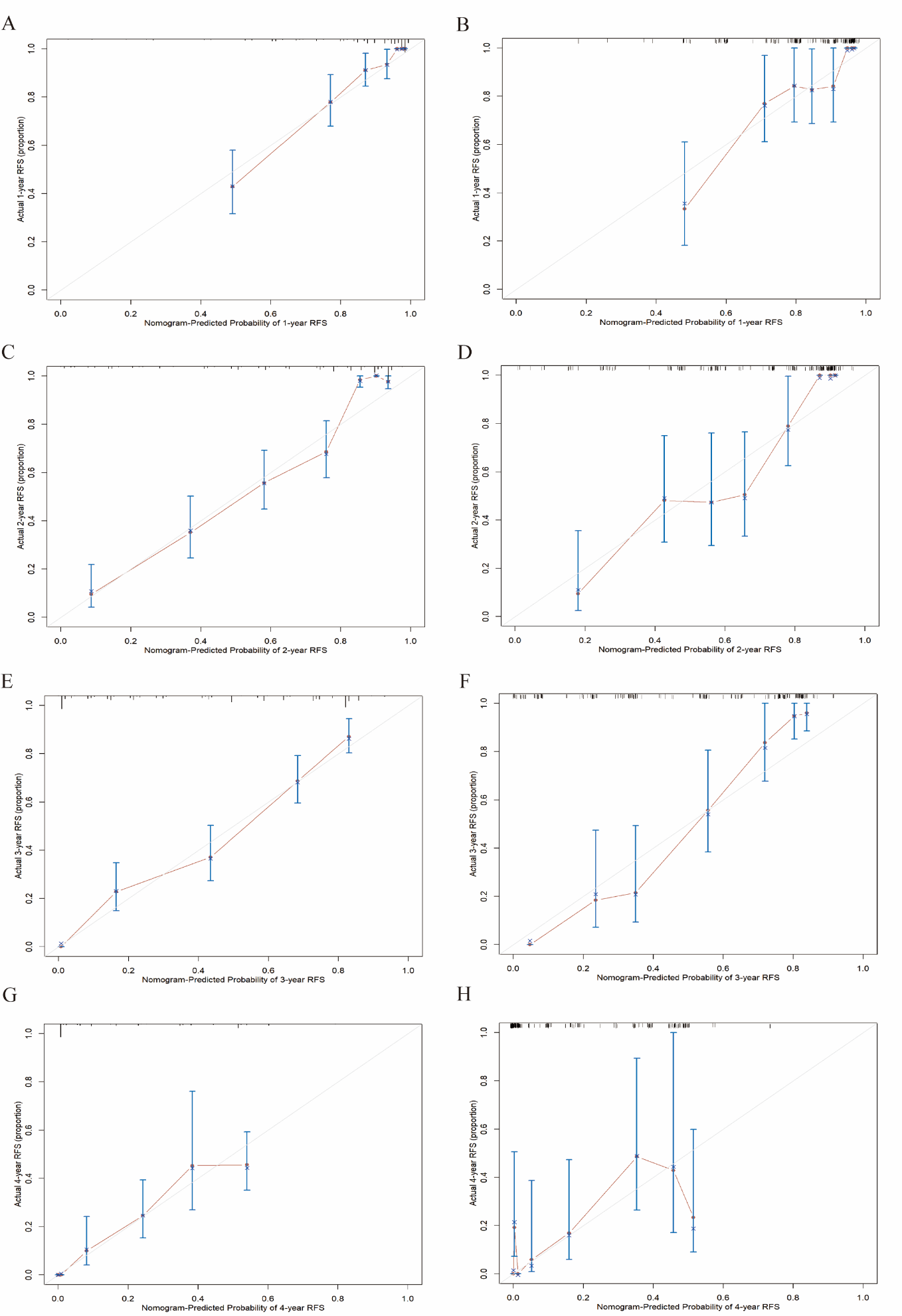


The x-axes are actual survival estimated by the nomogram; the y-axes are observed survival calculated by the Kaplan-Meier method. A, 1-year RFS in the training cohort; B, 1-year RFS in the validation cohort; C, 2-year RFS in the training cohort; D, 2-year RFS in the validation cohort; E, 3-year RFS in the training cohort; F, 3-year RFS in the validation cohort; G, 4-year RFS in the training cohort; H, 4-year RFS in the validation cohort.
